# Comparison of Biological Characteristics of Human Umbilical Cord Wharton’s Jelly-Derived Mesenchymal Stem Cells from Extremely Preterm and Term Infants

**DOI:** 10.1007/s13770-023-00538-9

**Published:** 2023-05-30

**Authors:** Peng Huang, Xiaofei Qin, Chuiqin Fan, Manna Wang, Fuyi Chen, Maochuan Liao, Huifeng Zhong, Hongwu Wang, Lian Ma

**Affiliations:** 1 Shenzhen Children’s Hospital of China Medical University, Shenzhen, 518038 China; 2 Affiliated Shenzhen Maternity and Child Healthcare Hospital, Southern Medical University, Shenzhen, 518028 China; 3 Department of Pediatrics, The Second Affiliated Hospital of Shantou University Medical College, Shantou, 515041 China; 4 Shenzhen People’s Hospital, Shenzhen, 518020 China; 5 Department of Pediatrics, The Women and Children’s Medical Hospital of Guangzhou Medical University, The Third Affifiliated Hospital of Guangzhou Medical University, Guangzhou, 510150 China

**Keywords:** HUMSCs, Extremely preterm infants, Neonatal diseases, HUVECs, Cell injury, TGFβ1

## Abstract

**Background:**

Despite the progress in perinatal-neonatal medicine, complications of extremely preterm infants continue to constitute the major adverse outcomes in neonatal intensive care unit. Human umbilical cord Wharton’s Jelly-derived mesenchymal stem cells (HUMSCs) may offer new hope for the treatment of intractable neonatal disorders. This study will explore the functional differences of HUMSCs between extremely preterm and term infants.

**Methods:**

UMSCs from 5 extremely preterm infants(weeks of gestation: 22^+5^ w,24^+4^ w,25^+3^ w,26 w,28 w) and 2 term infants(39 w,39^+2^ w) were isolated, and mesenchymal markers, pluripotent genes, proliferation rate were analyzed. HUVECs were injured by treated with LPS and repaired by co-cultured with HUMSCs of different gestational ages.

**Results:**

All HUMSCs showed fibroblast-like adherence to plastic and positively expressed surface marker of CD105,CD73 and CD90, but did not expressed CD45,CD34,CD14,CD79a and HLA-DR; HUMSCs in extremely preterm exhibited significant increase in proliferation as evidenced by CCK8, pluripotency markers OCT-4 tested by RT-PCR also showed increase. Above all, in LPS induced co-cultured inflame systerm, HUMSCs in extremely preterm were more capable to promote wound healing and tube formation in HUVEC cultures, they promoted TGFβ1 expression and inhibited IL6 expression.

**Conclusions:**

Our results suggest that HUMSCs from extremely preterm infants may be more suitable as candidates in cell therapy for the preterm infants.

## Introduction

With the progress of perinatal medicine, the survival rate of premature infants, especially extremely preterm infants (premature infants whose gestational age at birth is < 28 weeks or birth weight < 1,000 g) is increasing, but severe complications related to preterm infants are also increasing, such as bronchopulmonary dysplasia (BPD), periventricular-intraventricular hemorrhage (PVL-IVH), cerebral injury and necrotizing enterocolitis (NEC), which are still the main causes for death and disability of preterm infants [1–4] leading to long hospital stay and increased costs. Some of them will develop severe complications and even death. They may also suffer from the risks such as retarded growth and development, cerebral palsy after discharged. These sequelae can have impacts till adulthood, thus bringing a heavy burden to families and society. Therefore, it is an urgent and important task to develop new safe and effective treatment methods for improving the prognosis in preterm infants. In the past decades, the therapeutic application of mesenchymal stem cells (MSCs) in neonatal diseases has attracted many attentions [5–8].

Human umbilical cord mesenchymal stem cells (HUMSCs) possess the biological characteristics of MSCs. Comparing with bone marrow mesenchymal cells, they have higher proliferation capability, lower immunogenicity, no rejection in transplantation *in vivo*, as well as chemotaxis and migration to injured tissues. They can migrate to the injured site to play the role of immunoregulation and damage repair. They are easily available and do not cause harm to the human body. Compared with embryonic stem cells, they have no ethical and moral restriction. *In vitro* and *in vivo* studies have confirmed that HUMSCs have no tumorigenic risk and are safer than induced pluripotent stem cells (iPSCs). Therefore, they have been widely used in research on stem cell replacement therapy [9]. The therapeutic potential of HUMSC transplantation in preterm infant-related diseases has achieved certain effects in animal experiments, and its early clinical trials have been conducted in the prevention of BPD in premature infants and the treatment of severe IVH, which have also shown some effects [10–15].

There is evidence that the functions of stem cells may be related to the age. Duscher et al. have found that compared with young people, the migration capability and immunoregulatory capacity against inflammation of adipose-derived mesenchymal cells in the elderly reduce significantly [16]. Li et al. have revealed that MSCs will undergo epigenetic changes with passage (senescence), leading to their declined renewal ability and causing differentiation [17]. Nevertheless, Markel et al. have demonstrated that bone marrow MSCs in adults or the elderly can improve myocardial recovery after ischemia, while BMCs from newborns can not [18]. Consequently, the correlations between stem cells from different ages and their biological functions are still controversial. For newborns, there are different gestational ages at birth. It has been found that the content of hematopoietic stem cells in the umbilical cord blood of preterm infants is higher than that of term infants, and their cloning capability is higher. Later studies have confirmed that in the animal models of hypoxic-ischemic brain injury, the umbilical cord blood cells of premature and full-term infants can both normalize the white matter density and reduce cell death. However, their mechanisms are different: umbilical cord blood cells of preterm infants play a regulatory role by reducing tumor necrosis factor-α, while those of term infants alleviate oxidative stress by regulating the expression of interleukin (IL)-10 [19]. On this basis, the safety and feasibility of autologous umbilical cord blood cells in the treatment of cerebral injury have been confirmed by scientists in the United States and Japan, and phase I clinical trials have been completed [15, 20]. From 2020, Atul Malhotra’s team in Canada has intended to transplant autologous umbilical cord blood into extremely preterm infants under 28 weeks of gestation to prevent cerebral injury in premature infants [21]. Although it is convenient to obtain umbilical cord blood, for premature infants, especially extremely immature infants, delayed umbilical cord ligation can increase the blood volume and hemoglobin content of premature infants, reduce their occurrence of postnatal hypotension and anemia, and has many beneficial outcomes for long-term prognosis, thereby decrease the in-hospital mortality [22]. The 2019 European Guidelines for Management of Respiratory Distress Syndrome recommended delayed cord clamping (DCC) for at least 60 s for placental-fetal blood transfusion [23].

Therefore, compared with umbilical cord blood mesenchymal cells, HUMSCs can be used as ideal seed cells for autologous/allogeneic cell transplantation of preterm infants to replace precious umbilical cord blood cells. However, there is no deep understanding of the biological characteristics of HUMSCs in preterm infants, especially extremely preterm infants. We speculate that they have the same biological characteristics and functions as HUMSCs from term infants, and even have more advantages than those from term infants. Based on this, we collected the umbilical cord from 5 extremely preterm infants and 2 healthy term infants, and deeply explored the biological and functional differences of HUMSCs of different gestational ages, providing a new idea and basis for the future autologous/allogeneic HUMSC transplantation in the treatment of complications of preterm infants.

## Materials and methods

### Isolation and expansion of HUMSCs and HUVECs

This study was approved by the Institutional Review Board of the Second Affiliated Hospital of Shantou University Medical College. After written informed consent, umbilical cords from term (gestational age (GA) ≥ 37 weeks, n = 2, term) and extremely preterm (GA < 28 weeks or birth weight < 1000 g, n = 5) babies without intrauterine infection and congenital malformations were collected in a sterile environment. HUMSCs were isolated from umbilical cord as previously described [9]. The cells were cultured in fresh growth medium (containing the DMEM/F-12 with 10% fetal bovine serum) in a 37 °C humidified incubator with 5% CO2, when the media was replaced every 2 days. HUVECs were obtained from umbilical cord samples of healthy term infants using enzymatic digestion protocols described as Edyta [24]. The cells were cultured in M199 medium(Gibco, Grand Island, NY, USA) (containing 20% FBS and VEGF[Sciencell Research Laboratories, Carlsbad, CA, USA]) at 37 °C in 5% CO2 and used in passage 3–4.

### Flow cytometry phenotyping of HUMSCs

HUMSCs isolated from different gestation ages were characterized by flow cytometry in passage 3 or 4. Cells were washed in PBS for twice, then incubated in the dark with coupled mouse anti-human antibodies:FITC-CD90 (BioLegend, San Diego, CA, USA), PerCPCyTM5.5-CD105 (BD Biosciences, Heidelberg, Germany), APC-CD73 (BD Biosciences),PE-HLA-DR (BD Biosciences),PE-CD11b (BioLegend), PE-CD19 (BioLegend),PE-CD45 (BioLegend) and mouse anti-human PE-CD34 (BioLegend). After staining, the cells were washed twice with PBS and analyzed in a FACSCanto II cytometer (BD Biosciences).At least 20,000 cells were recorded, cells without adding any antibody as a negative control.

### Reverse transcription polymerase chain reaction and quantitative RT-PCR analysis

Total RNA was isolated from cells using the Eastep® super total RNA extraction kit (Promega, Madison, WI, USA) and 1-mg total RNA was used for reverse transcription using the GoScript™ reverse transcriptase (Promega). qRT-PCR was performed on a LightCycler 480 high flux Real-Time Fluorescence Quantitative PCR System (Roche, Indianapolis, IN, USA) using GoTaq ® qPCR Master Mix (Promega). PCR primer sequences are listed in Table 1 (Invitrogen, Shanghai, China).All the data were analyzed using GAPDH as an internal control. Relative copy numbers of target genes were determined using the 2-ΔΔCt method.

**Table 1:** List of primers used in RT-PCR

Gene	Gene primer sequence	Product size (BP)	Accession no
OCT4	F:GCAAAGCAGAAACCCTCGTG	169	NM_001173531.3
	R:CACACTCGGACCACATCCTT		
GAPDH	F:AATGGGCAGCCGTTAGGAAA	166	NM_001256799.3
	R:GCCCAATACGACCAAATCAGAG		

### Cell proliferation assay

Proliferation of HUMSCs was analyzed using cell counting kit-8 (CCK–8, Promega) according to the manufacturer’s protocols. HUMSCs were plated at a density of 2 × 10^3^ cells with 100ul medium into each well of a 96-well flat-bottom tissue culture plate (BD Falcon, San Jose, CA, USA),cultured for 4 h,24 h,48 h,72 h. CCK8 solution was added to each well and incubated in standard culture conditions for 1 h. The absorbance was analysed at 450 nm using a microplate reader (BioTek, Winooski, VT, USA), wells without cells as blanks. The results were expressed as mean absorbance in nanometers. All experiments were performed in triplicate.

### Cell injury model and co-culture system

HUVECs were seeded in 6-well plates and incubated for 12 h. The inflammatory model was treated with LPS(10 μg/ml). HUMSCs were pretreated with mitomycin for 24 h and then digested, 10^6^ cells were seeded on the upper layer chambers of Transwell plates (0.4 μm pore size; Corning Life Sciences, Tewksbury, MA, USA) at a ratio of HUVECs:HUMSCs of 2: 1. The supernatant was collected at 4 h, 24 h, and 48 h after co-culture, and the expression of inflammatory factor TGFβ1 was detected by Elisa. After 24 h’ co-culture, HUMSCs in the upper chambers were discarded. HUVEC in lower chambers were washed by PBS, and cultured in DMEM/FBS for Wound healing assay, or collected for the vasculogenesis assay.

#### ELISA

In co-cultured system, the supernatant was collected at 4 h, 24 h, 48 h and centrifuged (1000 × g, 5 min) to remove the cells. The levels of secreted TGF-β1and IL6 in the supernatant were measured according to the manual of the enzyme-linked immunosorbent assay (ELISA) kit(Human TGF-β1 elisa kit: Neobiosience, Shenzhen, China, EHC107b.96. AuthentiKine Human IL-6 ELISA Kit, Proteintech, USA. Human IL-1 beta ELISA Kit, Proteintech).The absorbance value at a wavelength of 450 nm was measured with a TriStar2 LB 942 Multimode Microplate Reader (Berthold Technologies, Bad Wildbad, Germany).

### Wound healing assay

LPS induced HUVECs were cultured with HUMSCs in transwell chambers(Tissue culture plate inserts, BIOFIL, Taipei, Taiwan) for 24 h, the upper chambers of HUMSCs were discarded, washed by PBS for twice,and cultured in DMEM/FBS for 24 h. scratch was made using a 200 μl pipette tip across the plates. After plates were washed with culture medium, HUVECs were grown for 24 h and photographs were taken.

### Tube formation

HUVECs were harvested after 24 h cocultured, and cells were (3 × 10^4^ cells per well) seeded onto matrigel plates (Matrigel356234; BD Biosciences) and cultured at 37 °C in 5% CO2. And observed by an inverted microscopy over the course of 24 h for tube formation. Networks formed by the HUVECs were quantified with Image J software (National Institutes of Health, Bethesda, MD, USA).

### Statistics analysis

All data were represented by mean ± standard deviations, and were analyzed by one-way ANOVA and Bonferroni’ multiple comparison test. Each result was completed by at least three independent experiments. The difference was statistically significant when *p* value < 0.05. Charts are drawn using GraphPad Prism(version 8.0 for Windows, GraphPad Software Inc., San Diego, CA, Bethesda, MD, USA).

## Results

### Clinical data of HUMSCs

In this study, umbilical cords from 7 neonates were collected for isolation and cultured of HUMSCs, and all of them were excluded from severe infection or congenital malformation. The clinical data of samples and outcomes of preterm infants show in Tables 2 and 3. There were 5 extremely preterm infants (birth weight < 1000 g) and 2 healthy full term infants (gestational age > 39 weeks), with a male to female ratio of 4:3,The minimum gestational age was 22^+5^ weeks, and the lowest weight was 498 g. Among the 5 preterm infants, 4 had histories of asphyxia, they needed long-term oxygen therapy and hospitalization (2–5 months), the morbidity of BPD was 100%, among which 3 cases were severe BPD (60%). And all of they had ROP. The baby born at 26 weeks was severe asphyxia and abandoned for treating by parents because of having grade III intracranial hemorrhage. These complications may have different degrees of influences on their growth and development after discharged and till adulthood. So an effective and safe therapy was needed to decrease the complications of these extremely preterm infants.

**Table 2:** Sample characteristics of HUMSCs

Sample	Gestational age (weeks)	Birth weight (g)	Mode of delivery	Apgar score 1 min	Apgar score 5 min	Sex
1	22^+5^	498	Vaginal	6	10	Male
2	24^+4^	595	Vaginal	7	10	Male
3	25^+3^	880	Vaginal	8	9	Male
4	26*	850	Vaginal	1	8	Female
5	28	990	Vaginal	5	9	Female
6	39	3020	Cesarean	10	10	Female
7	39^+2^	3470	Cesarean	10	10	Male

**Table 3:** Out comes of preterm infants

Sample	Gestational age (weeks)	Duration of mechanical ventilation (day)	Duration of oxygen therapy (day)	BPD	IVH	ROP	Duration of hospitalization (day)
1	22^+5^	48	104	Severe	N	Zone 2, Stage 1	111
2	24^+4^	34	157	Severe	N	Zone 2, Stage 3	157
3	25^+3^	1	37	Mild	I	Zone 2, Stage 3	85
4	26*	17	54	Severe	III	/	44
5	28	9	74	Moderate	N	Zone 2, Stage 1	78

### Wharton’s jelly isolation and HUMSCs culture

**Fig. 1:**
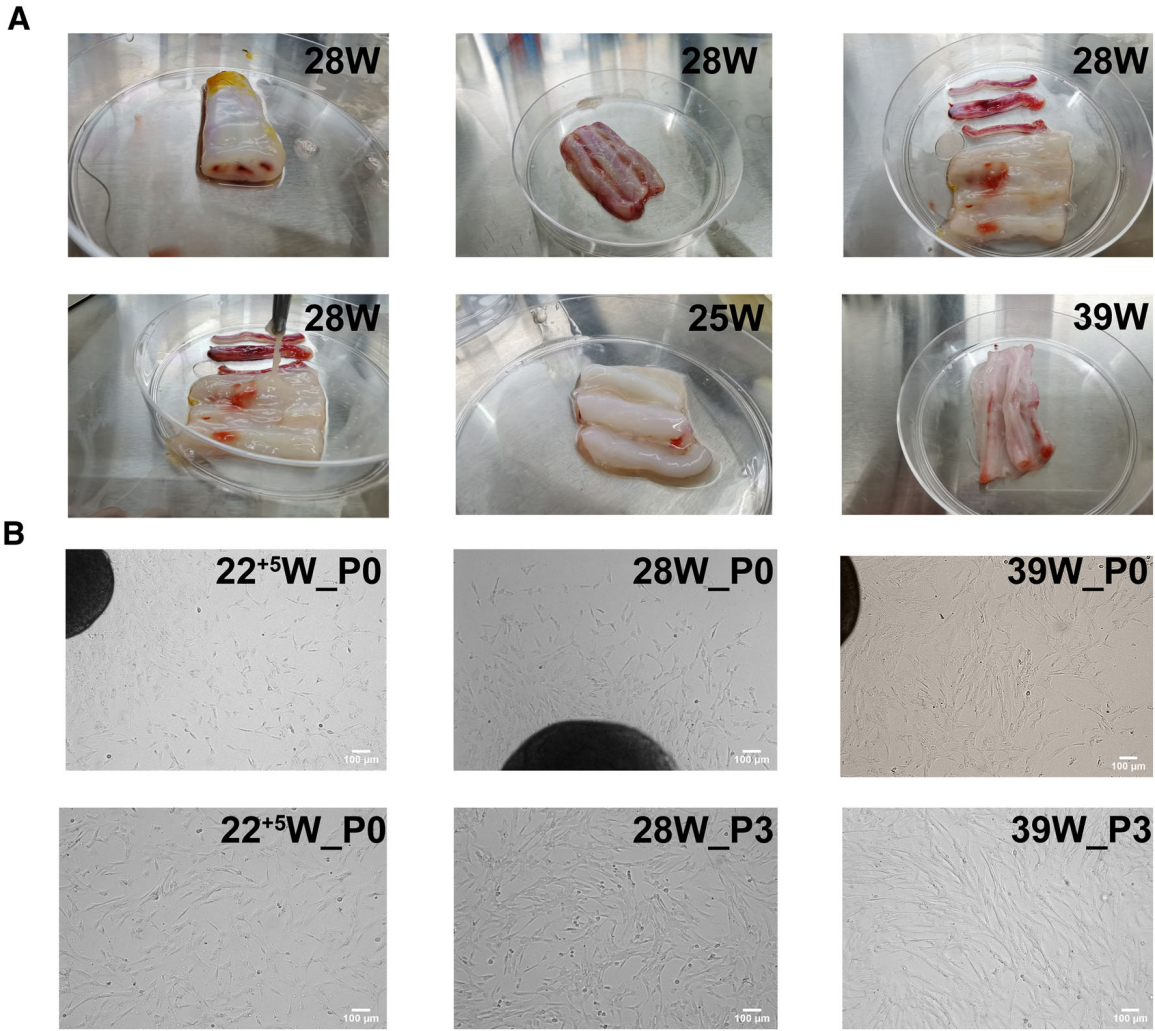
**A** Anatomical structure of human umbilical cord and Wharton’s jelly in different gestation ages, preterm infant had more jelly than term. **B** Morphology of HUMSCs in preterm and term infants after passage 3,there were no significant different between preterm and term in appearance. Magnification (× 100)

HUMSCs from 5 extremely preterm infants(weeks of gestation: 22^+5^ w, 24^+4^ w, 25^+3^ w, 26 w, 28 w) and 2 term infants(39 w,39^+2^ w) were isolated, when cutting Umbilical cord into pieces, we could clearly see the distribution of the umbilical vessels, tore amniotic carefully and removed the arteries an vein, the remaining tissue was Wharton’s jelly, we found that the cord in preterm infants contained more jelly than that in term infants(Fig. 1A).Wharton’s jelly was diced into small fragments and cultured in plates with fresh growth medium, when cells migrated out from jelly and reached 80–90% confluence, this is the passage 0 of HUMSCs, the durations of harvest P0 of HUMSCs were shown in Table 3, the P0 from small age of gestation needed a shorter time compared to elder gestation age (Table 4). HUMSCs were shown as fibroblast-like adhered cells, and there was no significant difference between preterm and term in appearance (Fig. 1B).

**Table 4:** Duration of primary culture in different gestation age of HUMSCs (P0)

Gestation age (week)	22^+5^	24^+4^	25^+3^	26	28	39	39^+2^
Time (day)	3	3	4	4	6	7	7

### MSC surface marker and pluripotency Marker expression

**Fig. 2:**
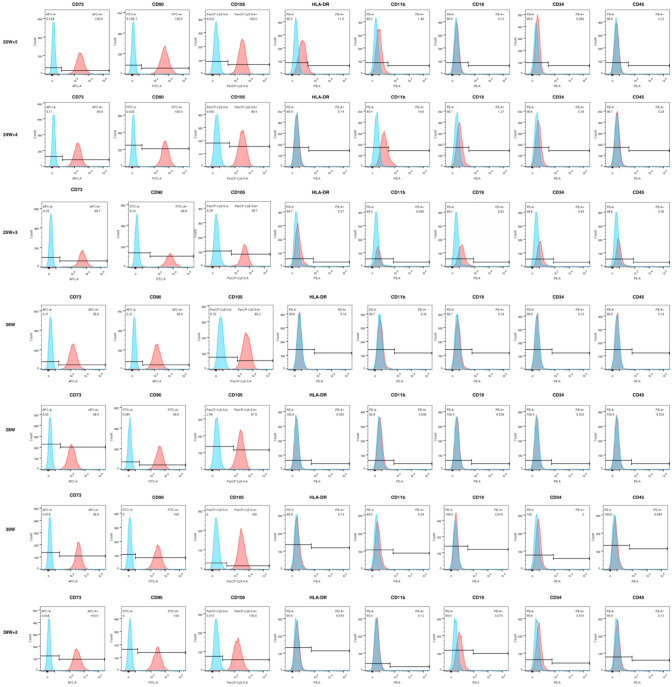
MSC surface marker of HUMSCs in different gestation ages by flow cytometer. HUMSCs in preterm and term infants were positive for CD105, CD73 and CD90, but negative for CD34,CD14 (CD11b),CD79a(CD19), CD45 and HLA-DR)

**Fig. 3:**
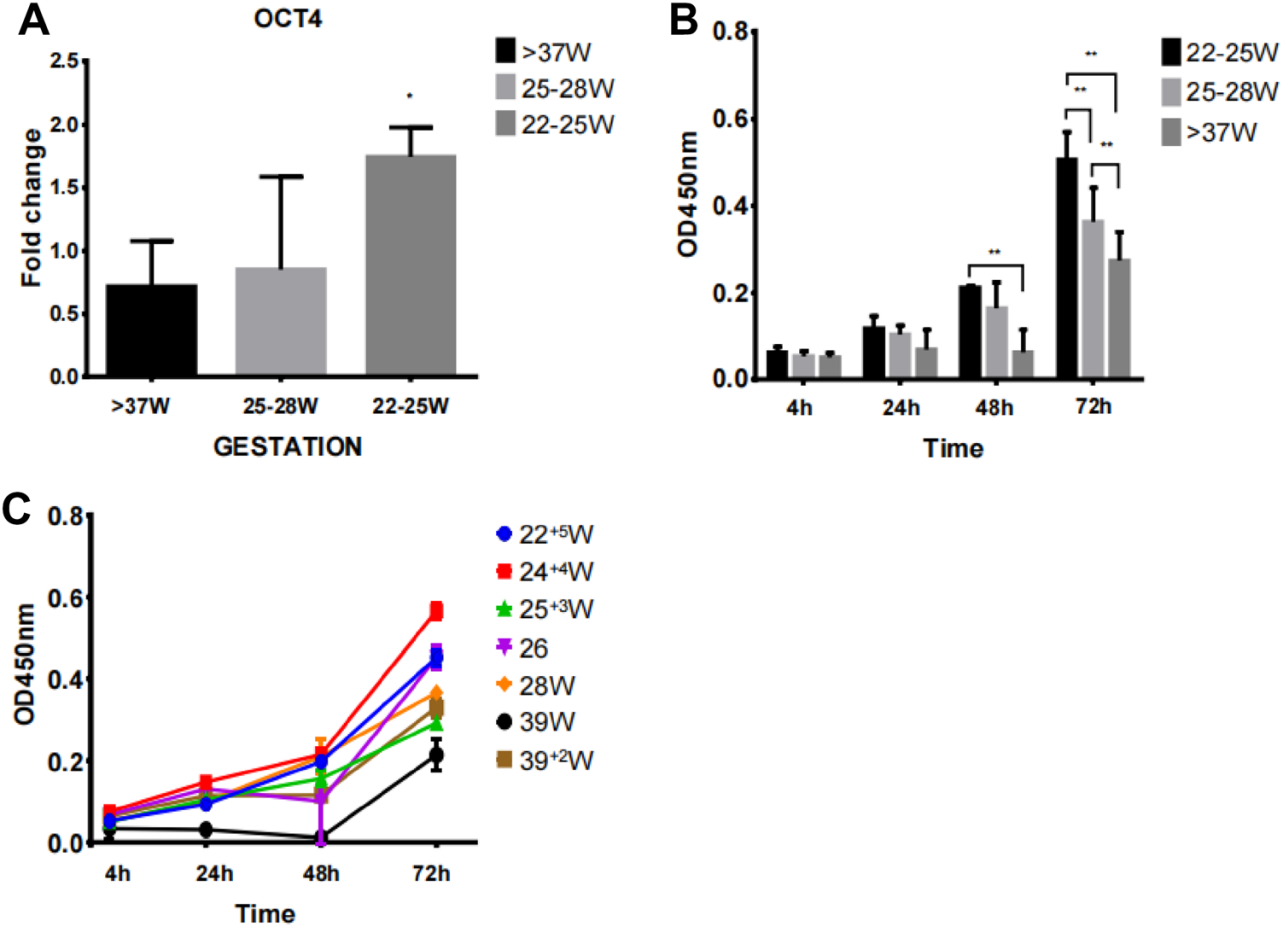
**A** Expression of OCT4 in preterm and term HUMSCs by quantitative RT-PCR,OCT4 expressed in 22-25w was higher than that in term,and the difference was significantly(*: *p* < 0.05).**B** Proliferation of different gestation age HUMSCs in different time(4 h,24 h,48 h,72 h), HUMSCs in preterm had a higher proliferative potential. **C** Growth curve oh HUMSCs in preterm and term infants

HUMSCs from preterm and term in passage 3 or 4 were characterized for the expression of cell surface markers using flow cytometer, and both preterm and term HUMSCs were positive for MSC signature markers (CD105, CD73 and CD90) but negative for endothelial(CD34) and monocyte/macrophage(CD14 or CD11b) and hematopoietic (CD79a or CD19 and CD45), major histocompatibility complex(HLA-DR) markers (Fig. 2). So their surface marker characterizations matched the minimal criteria for defining MSCs established by the ISCT. HUMSCs were considered possessing the multipotent potential including expressing of pluripotency markers such as OCT-4, SOX-2 and NANOG,however, the “stemness” in HUMSCs was controversial as reported from literature. We detected the expression of OCT4 in mRNA level by RTPCR in different gestation ages of HUMSCs. The results shown that expression of OCT4 decreased as gestation proceeded to term, and 22–25wk HUMSCs were significantly high expressing compared to 25–28wk and term infants (Fig. 3A).

### Proliferation assay by CCK8

The CCK8 assay was performed to investigate the proliferation of different gestation ages of HUMSCs in passage 3. The results showed that the proliferation rates of HUMSCs in 22–25w, 25–28w and term increased as the culture time increased(Fig. 3C), at the time point of 4 h and 24 h, the OD values were similar between groups, but in 48 h, 22–25w had a significant higher proliferation rate contrasted to 25–28w and term, in 72 h, both preterm groups had a significant higher proliferation than term(Fig. 3B), indicated that HUMSCs in preterm infants have markedly higher capability of proliferation than that of term infants.

### Isolation of HUVECs and LPS induced cell injury *in vitro*

**Fig. 4:**
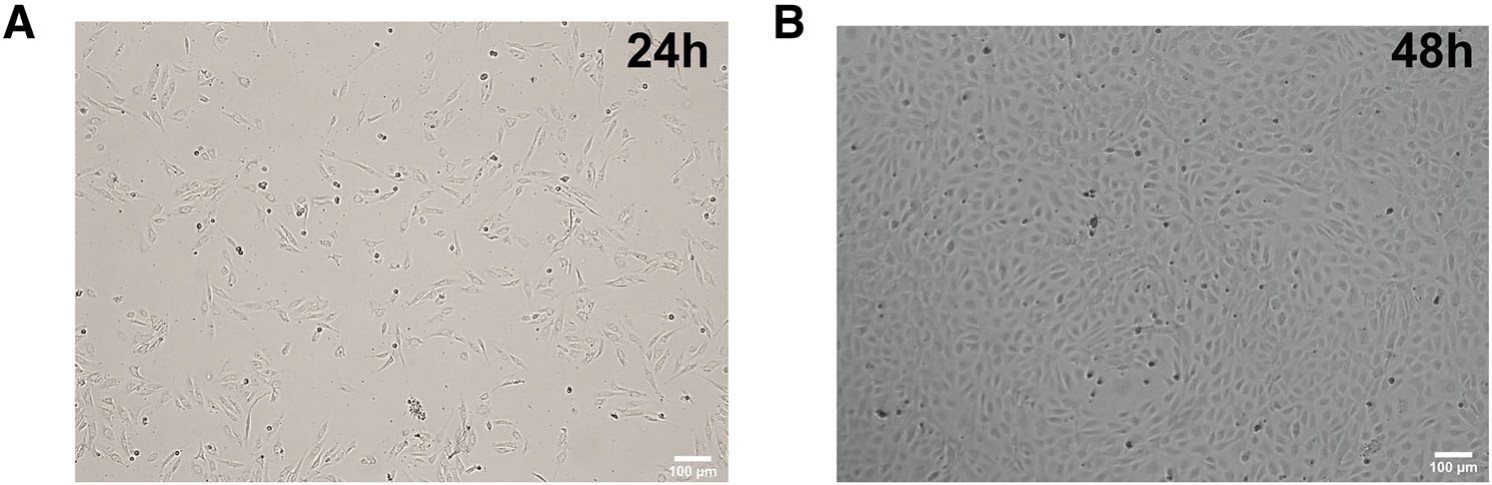
**A**, **B** Morphology of HUVECs of primary culture in 24 h and 72 h, the typical cobblestone morphology of HUVECs shown in **B** Magnification (× 100)

Human umbilical vein endothelial cells digested with trypsin were seeded in a 10 cm cell culture dish for about 4 h, most of the cells began to adhere and showed pleomorphism, but smaller than HUMSCs. After 24 h (Fig. 4A), the cells gradually grew into spindle, and some of them were similar to fibroblasts. At 48–72 h, the cells grew fast, and soon reached confluence, exhibited the typical cobblestone morphology, that is the characteristic of endothelial cells(Fig. 4B). HUVECs were incubated with LPS(10 μg/mL) for 4, 24, or 48 h to induce endothelial injury. In the mean time, HUMSCs were seeded in the upper chambers of Transwell plate for cocultrue with the injury HUMSCs.

### HUMSCs coculture with HUVEC and repaired cell injury

**Fig. 5:**
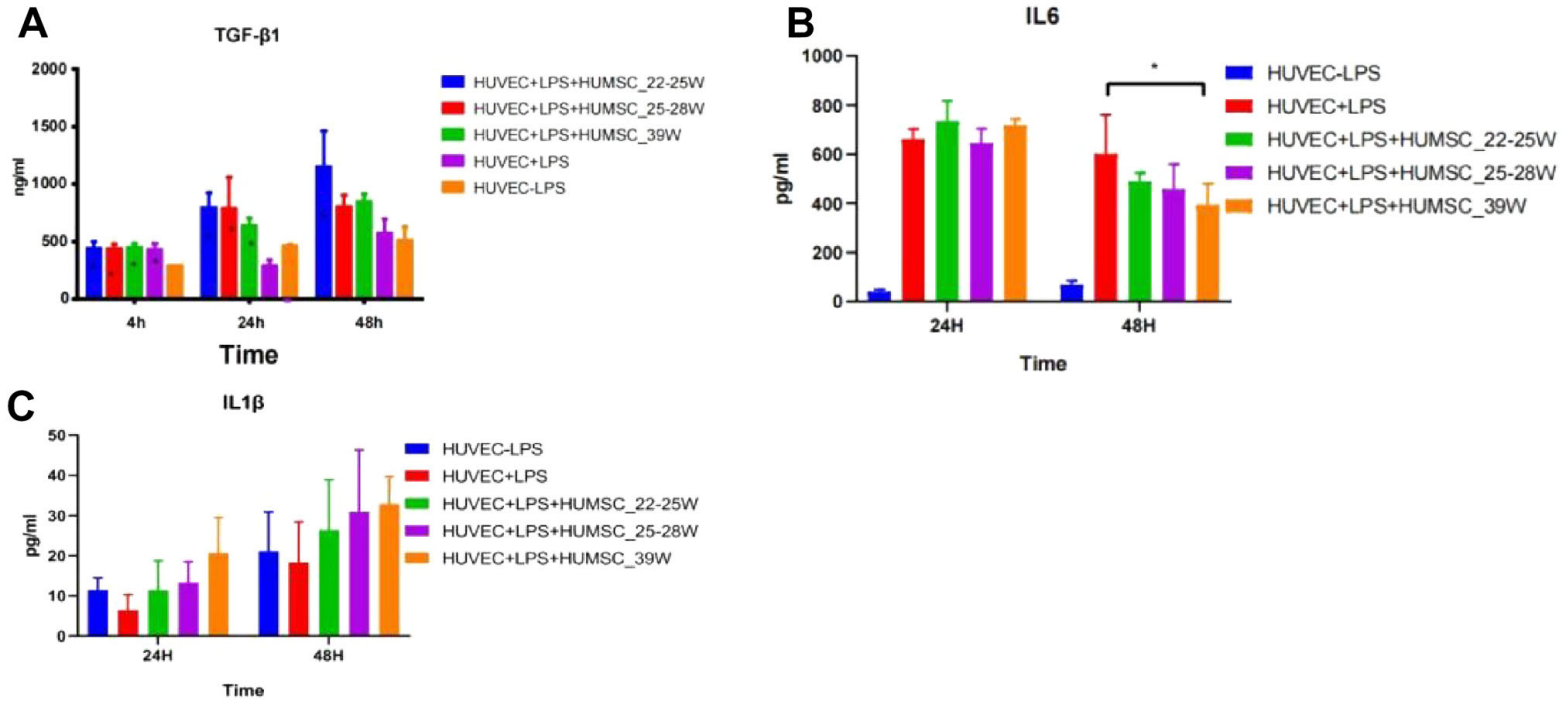
Inflammatory factors of **A** TGFβ1, **B** IL6 and **C** IL1β secreted in LPS induced of HUVECs and co-culture with or without HUMSCs in different gestation ages in different time by ELISA.HUVEC + LPS + HUMSC_22-25w:HUVEC induced by LPS and co-culture with HUMSCs of 22-25 weeks gestation age, HUVEC + LPS:LPS induced HUVEC without co-culture with HUMSCs. HUVEC-lps: HUVEC did not induce by LPS and also did not co-cultrue with HUMSCs, and was considered as the control group in statistical analysis,*:*p* < 0.05

To explore the immune suppressive HUMSCs of different gestation ages in inflammation,we first collected medium after coculturing at the time point of 4 h.24 h, and 48 h, the inflammatory factor TGFβ1,L6 and IL1βwere detected by ELISA. as shown in Fig. 5A. TGF β1 secret increased significantly after 4 h LPS induced HUVECs,and after 24 h, concentration of TGF β1 secret in HUVECs cocultured with HUMSCs were higher than that of without HUMSCs, in 48 h, HUVECs cocultured with HUMSCs of 22–25W were high secret TGF β1 compared to 25–28w and term. these mean that HUMSCs promoted TGF β1 secreting to anti-inflammation in inflammation environment, especially in the HUMSCs from extremely preterm infants. Figure 5B demonstrates that LPS-induced inflammation led to a marked increase in IL6 secretion by HUVECs after 24 h. Despite the copious expression of this proinflammatory cytokine, co-culture with HUMSCs did not significantly alter IL6 levels. Notably, after 48 h, HUMSCs exhibited a significant reduction in IL6 expression compared to HUVECs alone. This downregulation was particularly pronounced in term HUMSCs, as evidenced by a significant intergroup difference. However, statistical analysis revealed no significant differences between HUVECs co-cultured with preterm or term HUMSCs. In addition to IL6, we assessed the expression of IL1β, another key inflammatory factor, as shown in Fig. 5C. Notably, at both the 24 and 48 h timepoints following LPS induction, the levels of IL1β were low and did not exhibit significant differences between the groups with and without LPS treatment. This finding indicates that HUVECs are unable to robustly upregulate IL1β expression in response to inflammation.

**Fig. 6:**
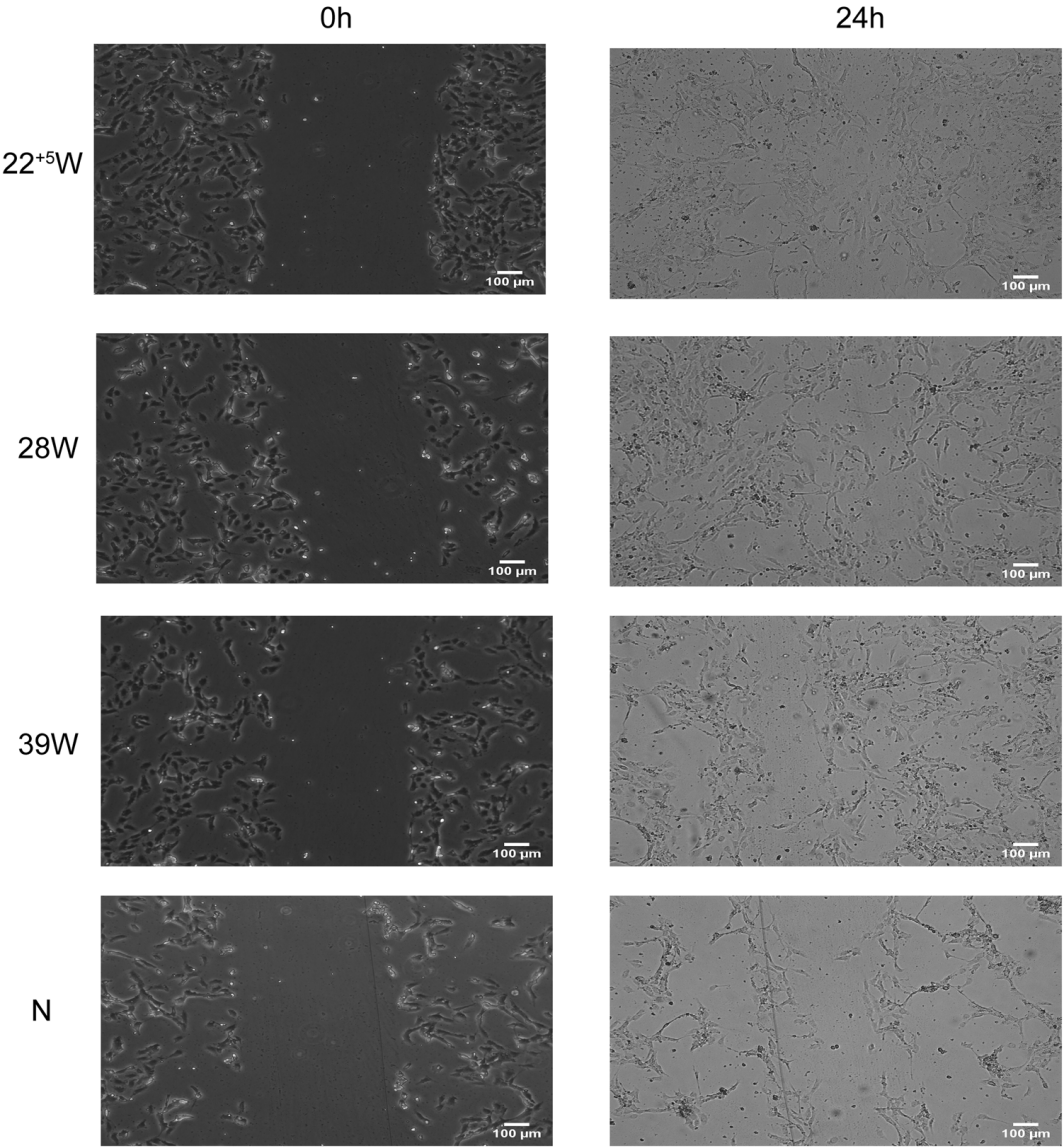
Wound healing assay: HUVEC were induced by LPS and co-culture with or without HUMSCs for 24 h, after that, HUMSCs were removed, monolayers of HUVEC were wounded, Pictures were taken at the time of wounding and 24 h later. Magnification (× 100)

To test migration, we performed scratch wound assays on LPS induced of HUVECs. We then monitored the migration of each cell group into the wound in the coculture with that of different gestation ages of HUMSCs. As shown in Fig. 6. LPS induced HUVECs without coculturedwith HUMSCs has a low concentration after 24H,and the cells migrated into the scratc area significantly slower than that of coculture with HUMSCs,there was an increase in migration of HUVECs correlated with the gestational ages of HUMSCs, preterm HUMSCs were more capable to increasethe would healing in injured HUVECs.

**Fig. 7:**
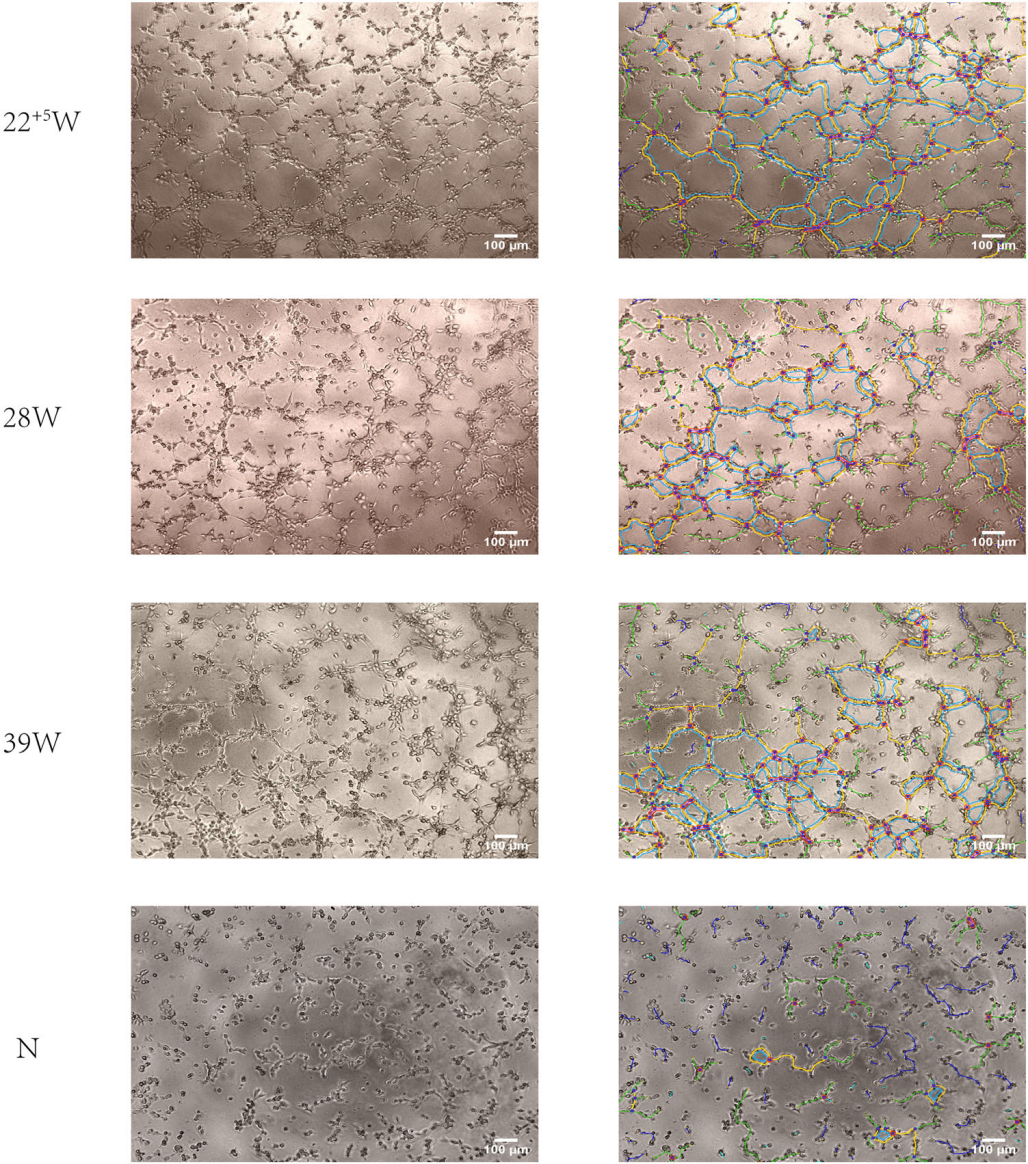
HUVEC were induced by LPS and co-culture with or without HUMSCs of different gestation ages, after 24 h, HUVEC were collected and plated on Matrigel. Cells were monitored for capillary morphogenesis and photographs were taken after 24 h. Magnification (× 100)

To determine the effect of HUMSCs from different gestation ages in mediating injured HUVECs repairing, LPS induce HUVECs was cocultured with or without HUMSCs, were collected; then, tube formation of HUVECs was assessed using the Matrigel assay, the tube was quantified using Image J software. As shown in Fig. 7 compared with the negative control(coculture without HUMSCs), HUVECs cocultured with HUMSCs had obvious proangiogenic effects at 24 h, and the angiogenic effects from preterm HUMSCs were more stronger.

## Discussion

During the primary culture of HUMSCs from these umbilical cords of different gestational ages, we found that Wharton’s jelly in the umbilical cord of premature infants seemed to have a higher content than that of term infants, and was easier to obtain. Therefore, it can ensure enough tissue for culture, which is very beneficial for preterm infants. Early application of MSCs can prevent the occurrence of preterm infant-related complications such as BPD, NEC, ROP and IVH, or reduce the severity of the diseases [25, 26]. In this respect, collecting HUMSCs from preterm infants is more convenient and earlier than that from term infants. Our study also confirmed that in both primary culture and culture after passage, the viability and proliferation of HUMSCs from preterm infants were superior to those from term infants. Our results preliminarily demonstrated that HUMSCs cultured from the umbilical cord of extremely preterm infants had the same MSC characteristics as term infants, and fulfilled the standards of pluripotent mesenchymal stem cells formulated by the International Society for Cellular Therapy (ISCT) [27]: fibroblast-like adherent growth under standard culture conditions; high expression of CD29, CD44, CD73, CD90 and CD105, but no expression of CD45, CD34, CD19, CD31 and HLA-DR. In our experiment, the analysis of RNA levels showed that the expression of OCT4 in HUMSCs of 22–25 gestational weeks was higher than that in term infants, suggesting that these HUMSCs from preterm infants are lowlily differentiated and may have a stronger multi-directional differentiation potential.

At the early stage after the proposal of MSCs, people mainly focused on their multi-directional differentiation potential and expected that they could be used for cell transplantation in tissue regeneration. However, this view is no longer universally recognized [6]. With people’s in-depth understanding of MSCs, it has been found that the therapeutic effect of MSCs is mainly mediated by the paracrine mechanism, and their therapeutic effect on body damage may be achieved by activating some endogenous repair pathways [28, 29]. Numerous studies have confirmed that MSCs can secrete a series of growth factors and cytokines, such as vascular endothelial growth factor, keratinocyte growth factor, stem cell growth factor, IL-6, IL-10 and IL-11, which can play a direct immunomodulatory role [30]. In addition to secreting these cytokines and growth factors, MSCs also secrete extracellular vesicles (EVs). EVs are membrane-bound carriers with complex components, including proteins, lipids and nucleic acids, which can transmit signals to other cells and change the functions of these target cells [31, 32]. In 2009, Aslam et al. found that MSC-derived conditioned medium (MSC-CM) can improve hyperoxia-induced pulmonary injury in animal models of BPD. Compared with direct transplantation of MSCs, MSC-CM transplantation presents a more obvious protective effect on pulmonary structure, which strongly supports the paracrine effect of MSC therapy [33]. In recent years, the focus on MSC-CM has presented a straight upward trend. It has been found that MSC-CM contains various growth factors and immunoregulatory factors secreted by cells, as well as EVs. Therefore, cell-free MSC derivatives are also proposed [34, 35]. The components of MSC-CM will vary with environmental changes, so external stimulation is needed for secreting relevant cell components. It has been proved in animal experiments that in different animal models, MSCs secrete different cytoprotective factors under different environments. In the animal model of BPD, human vascular endothelial growth factor (VEGF) secreted by transplanted human MSCs induces the expression of VEGF in the host hyperoxic lung [36]. In the animal model of IVH, human brain-derived neurotrophic factor (BDNF) secreted by transplanted human MSCs plays an important role in MSC-induced neural protection [37]. In our study, the adopted co-culture could make the therapeutic cells in a specific environment, so as to accurately exert their role. Indeed, we observed that HUMSCs were beneficial to HUVECs with inflammatory injury. The migration and angiogenic capability of co-cultured HUVECs could be maintained under the inflammatory environment, which is speculated to be related to that they can up-regulate the expression of transforming growth factor TGFβ1, and Inhibited LPS-Induced elevation of Pro-inflammatory Cytokines IL6. TGFβ1 is a member of the TGFβ superfamily that can regulate cell growth and differentiation. TGFβ1 as an anti-inflammatory factor, it plays a variety of roles in various physiological and pathological environments, such as playing an important role in angiogenesis, and promoting cell migration and maintainning the angiogenic function of HUVECs [38]. Compared to term infants, we found that TGFβ1 expression in the inflammatory environment was strongly promoted by HUMSCs of extremely preterm infants, but its specific mechanism remains to be clarified in our future work.

In this study, only HUMSCs from 5 extremely preterm infants and 2 term infants were selected as the subjects, preterm infants of other gestational ages were not included because of refractory complications which is relatively low in preterm infants with a gestational age of more than 28 weeks In addition, preterm infants with intrauterine infection and developmental malformation were excluded from the samples we selected. As a result, the sample size is insufficient. We hope that more researchers will pay attention to the research on HUMSCs from preterm infants and further explore their biological characteristics. In this study, we observed that HUMSCs effectively downregulated the expression of IL6 in HUVECs under LPS-induced inflammation. However, the extent of this downregulation did not significantly differ between term and preterm HUMSCs. In addition, we did not detect IL1β high expression in HUVECs in response to LPS induction. This finding suggests that HUVECs may not produce IL1β in the context of inflammation. We hypothesize that HUMSCs did not fully suppress all inflammatory factors, or additional immune cell types, such as megakaryocytes, may be involved in modulating inflammatory responses. Further investigation is needed to elucidate the full extent of HUMSCs’ anti-inflammatory capabilities and their mechanisms of action. In conclusion, our study preliminarily confirms that HUMSCs from extremely preterm infants have higher proliferation capability and pluripotency than those from term infants and a more significant repairing effect on damaged cells. Therefore, they may be an ideal seed cell source for cell therapy to treat refractory complications of preterm infants.
